# Utility of the chromogenic and fluorogenic properties of benzofurazan for the assay of milnacipran in human urine and plasma†

**DOI:** 10.1039/c8ra03614d

**Published:** 2018-06-15

**Authors:** Islam M. Mostafa, Mahmoud A. Omar, Dalia M. Nagy, Sayed M. Derayea

**Affiliations:** Analytical Chemistry Department, Faculty of Pharmacy, Minia University Minia Egypt dr-islam@mu.edu.eg

## Abstract

Our article presents the development and validation of two simple, very sensitive, and low-cost spectroscopic methods for the assay of milnacipran hydrochloride in bulk form, pharmaceutical tablets and spiked human urine and plasma. Spectroscopic methods (spectrophotometric and spectrofluorimetric techniques) were dependent on the chromogenic and fluorogenic properties of the 4-chloro-7 nitrobenzofurazan (NBD-Cl) reagent. The reaction product, resulting from the interaction between NBD-Cl and milnacipran in the presence of borate buffer pH 8.5, was measured spectrophotometrically at 465 nm and spectrofluorimetrically at 510 nm after excitation at 465 nm. The absorbance–concentration plot was rectilinear over the range of 1.5–12 μg mL^−1^ with a limit of quantitation 1.09 μg mL^−1^, while the fluorescence–concentration plot was rectilinear over the range of 0.03–0.5 μg mL^−1^ with a limit of quantitation 0.02 μg mL^−1^. Influential parameters affecting the development and stability of the reaction product were studied and optimized. Assurance of the cited drug in its tablets by our proposed methods was successfully completed without obstruction from the presence of the basic excipients with average percentage recoveries of 99.27 ± 1.18 and 99.44 ± 0.69 for the spectrophotometric and spectrofluorimetric methods, respectively. The spectrofluorimetric method was additionally adopted as a preliminary *in vitro* study for the assay of the cited drug in spiked human urine and plasma with average percentage recoveries of 101.52 ± 1.01 and 100.38 ± 1.57 for spiked urine and plasma, respectively.

## Abstract 2

Our article presents the development and validation of two simple, very sensitive, and low-cost spectroscopic methods for the assay of milnacipran hydrochloride in bulk form, pharmaceutical tablets and spiked human urine and plasma.

## Graphical abstract

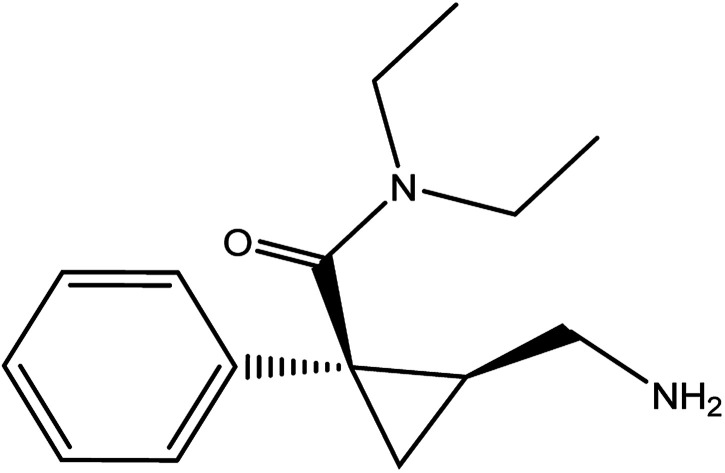

## 1. Introduction

**Fig. 1:**
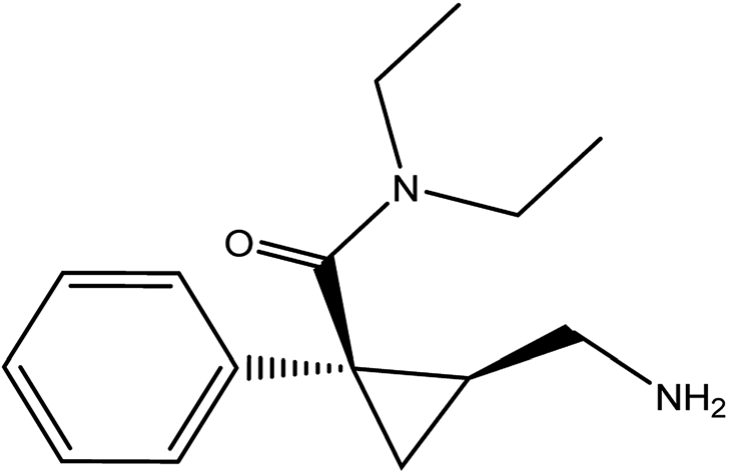
Chemical structure of milnacipran hydrochloride.

Milnacipran hydrochloride (MCH) is used for the treatment of depression in some countries due to its action as a serotonin and noradrenaline reuptake inhibitor.^1,2^ Its oral dose is 50 mg twice daily.^3^ MCH is mainly used in the treatment of fibromyalgia^4^ which is characterized by widespread pain and decreased physical function. It is chemically named as (±)-*cis*-2-(aminomethyl)-*N*,*N*-diethyl-1 phenyl cyclopropane carboxamide hydrochloride (Fig. 1). Analytical investigation of MCH in biological samples as well as tablets was reported by spectrophotometric^5,6^ and chromatographic methods.^7–16^

Only two methods were reported for the determination of MCH. The first one was based on measurement of the absorbance of the drug solution at 220 nm. This low wavelength makes the method liable for interference even from some solvents. The second method overcame this limitation through the reaction of the dug with ninhydrin to give purple color that could be measured at 570 nm. However the later method was tedious as it required heating at elevated temperature (120 °C) on a glycerol bath. In addition both methods have low sensitivity (LOQ were 1.61 and 1.67 μg mL^−1^).^5,6^ Moreover, chromatographic methods^7–16^ required sophisticated instrumentation and consume large volumes of the organic solvents which elevate the cost of the analysis and have bad impact on the environment. Although spectrofluorimetry is a simple technique with high sensitivity, unfortunately no spectrofluorimetric method was reported for the determination of MCH in any matrices. In the current study derivatization with NDB-Cl reagent was utilized as chromogenic and fluorogenic probe for the determinations of the cited drug with a simple procedure. The proposed methods are more sensitive than the reported spectrophotometric method (LOQ; 1.09 and 0.02 μg mL^−1^ for the spectrophotometric and spectrofluorimetric methods, respectively) and low cost. Herein, the present study represents the first spectrofluorimetric method for determination of MCH. The proposed spectroscopic methods can be easily used in quality control laboratories for determination of the studied drug in commercial tablets as well as spiked human urine and plasma.

## 2. Experimental

### 2.1. Apparatus

All the measurements of the two proposed methods were done by using 1-Spectronic™ Genesys™ 2 PC UV/Visible Spectrophotometer (Milton Roy Co, made in USA).

2-LS 45 spectrometer device (company; Perkin Elmer, made in UK) enriched with quartz cell (1 cm) and xenon lamb (150 watt). Two monochromators were used with opening width 10 nm for both. FL WINLAB™ program was installed in personal computer for operating the spectrometer.

### 2.2. Material and chemicals

Standard authentic MCH and Averomilan® pharmaceutical tablets (each tablet contains 50 mg of MCH) were obtained from Averroes Pharma for pharmaceutical industries (Cairo, Egypt) as a gift. Benzofurazan (NBD-Cl, Sigma Aldrich for chemicals, St. Louis, USA) was freshly prepared as 1 mg mL^−1^ in methanol. Borate buffer, 0.1 M of different pH values was prepared. Boric acid, hydrochloric acid, acetone, sodium hydroxide, methanol and acetonitrile were purchased from (El-Nasr Co for Intermediate Chemicals, Egypt).

### 2.3. Standard solution preparation

Preparation of the standard MCH solution was done by dissolving 10.0 mg in 100 mL methanol. Then the working experimental solution of MCH was prepared by further dilution of the standard stock solution to obtain different solutions in the concentration range of 15–120 μg mL^−1^ and 0.3–5 μg mL^−1^ for the spectrophotometric and spectrofluorimetric method, respectively.

### 2.4. Analytical procedure for construction of the calibration curves

#### 2.4.1 For spectrophotometric method

Into a thermostatically controlled water bath adjusted at 70 °C, test tubes were added which contained 1.0 mL of MCH (15–120 μg mL^−1^), 1.0 mL of borate buffer solution (pH 8.5) and 0.5 mL of NBD-Cl (0.1% w/v) for 20 min. After that, the heated solutions in the test tubes were cooled by using an ice bath. Then the content of each test tube was acidified with 0.2 mL of conc. hydrochloric acid. Finally, the content of the test tubes were mixed well and moved quantitatively to 10.0 mL calibrated flask then completed to the volume with acetone. The absorbance was estimated at 465 nm against a reagent blank treated with the same manner.

#### 2.4.2 For spectrofluorimetric method

Spectrofluorimetric method was carried out in the same manner as spectrophotometric method except the drug solution was in the concentration range of 0.3–5.0 μg mL^−1^ and the emission intensity of the yellow fluorescent product was estimated at 510 nm (*λ*_ex_ 465 nm). At the same time a blank experiment was estimated similarly without adding the MCH drug solution.

### 2.5. Assurance of MCH in Avermilan® pharmaceutical tablets

Ten Avermilan® tablets were weighed and powdered, and then into 100 mL volumetric flask a precise weight equivalent to 10 mg MCH was transferred, mixed with 25 mL of methanol and sonicate for a 30 min and afterward the volume was completed to 100 mL methanol and was filtered. Large part of the filtrate was disposed and 1.0 mL of the filtrated solution was diluted to 10 mL with the same diluent. One concentration found in the linear range of the two spectroscopic methods was moved into test tubes and analyzed as previously mentioned under Section 2.4.

### 2.6. Assay of MCH in spiked human urine and plasma by the proposed spectrofluorimetric method

All experiments were performed in compliance with the relevant laws and institutional guidelines (Credibility and Ethics guidelines, Minia University, Egypt), and the academic committee of Minia University has approved the experiments. In all cases, informed written consent was obtained from all participants.

#### 2.6.1. Procedures for spiked plasma

Into heparinized blood test tube, free blood samples was acquired from healthy volunteers and centrifuged at 4000 rpm for 30 minutes. Into a centrifuge tube, add 1.0 mL of the free plasma which was spiked with 1.0 mL standard MCH (3–50 μg mL^−1^). The content was completed to volume 10.0 mL with methanol (which also acts as a precipitating agent for proteins) to obtain a concentration of 0.3–5 μg mL^−1^. After that the solution was put into centrifuge for around 30 min at 4000 rpm. Different volumes of the acquired supernatant were taken for their assay by the general procedure of spectrofluorimetric method. At the same time of the analysis of the plasma samples, a blank was estimated by applying the same steps on a blood sample without drug.

#### 2.6.2. Procedures for spiked urine

One milliliter of human urine was spiked with 1 mL of MCH standard authentic solution (50 μg mL^−1^), then 2 mL methanol was added and the content was diluted to 10 mL with distilled water, then centrifuged at 4000 rpm for 10 min. Into a 10 mL glass tubes three different volumes of the clear supernatant (5 μg mL^−1^) equivalent to 1.0, 3.0 and 5.0 μg mL^−1^ were transferred and the general procedure for the proposed spectrofluorimetric method was followed.

## 3. Results and discussion

**Fig. 2:**
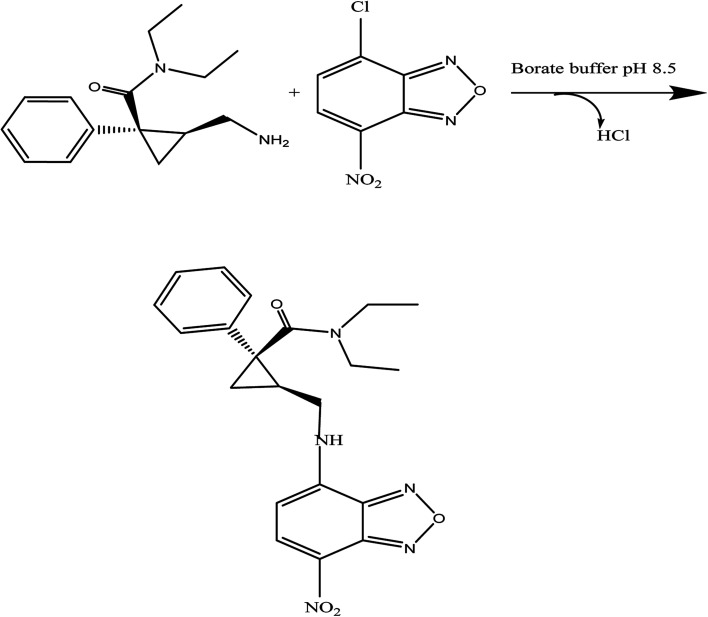
The proposed mechanism for the reaction between MCH and NBD-Cl in borate buffer (pH 8.5).

**Fig. 3:**
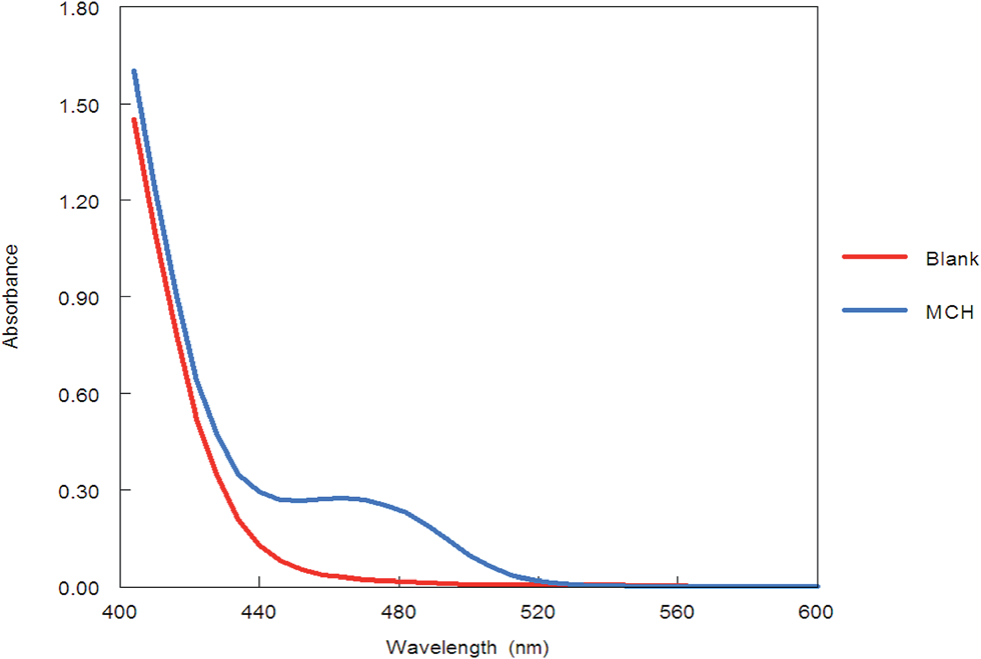
Absorption spectra of the reaction product between NBD-Cl and MCH (5.0 μg mL^−1^).

**Fig. 4:**
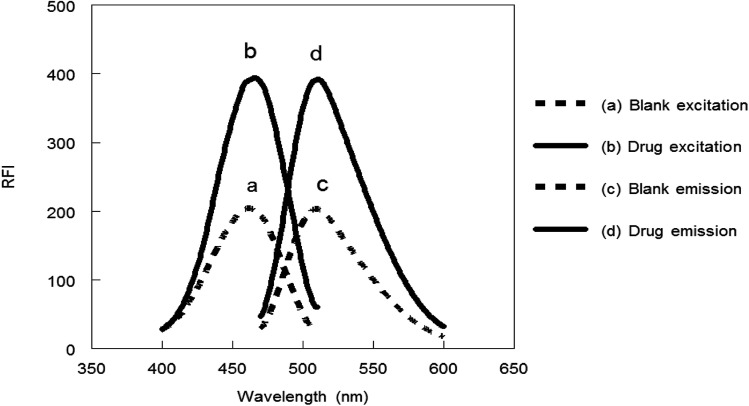
Excitation and emission spectra of the reaction product between NBD-Cl and MCH (0.1 μg mL^−1^).

Benzofurazan reagent is characterized by its chromogenic and fluorogenic properties that can be used for the derivatization of primary, secondary amines compounds and alcoholic hydroxyl compounds. Many pharmaceutical compounds^17–19^ were determined spectrophotometrically and/or spectrofluorimetrically by utilizing chromogenic and fluorogenic properties of NBD-Cl. The interaction between NBD-Cl with compounds bearing basic primary, secondary amino group or hydroxyl group resulted in the formation of colored and/or fluorescent product. In our study, the interaction of the amino group of MCH with NBD-Cl in borate buffer pH 8.5 was carried out for the first time and it is also considered the first spectrofluorimetric method for analysis of MCH in its tablets and spiked human urine and plasma. The reaction pathway is postulated as appeared in Fig. 2. The yellow colored product displayed its absorbance at *λ*_max_ 465 nm (Fig. 3) as well as its powerful emission fluorescence intensity at *λ*_em_ 510 nm after excitation at *λ*_ex_ 465 nm (Fig. 4). Notably, the drug and the reagent have no absorbance or fluorescence properties at the measured wavelengths.

### 3.1. Optimization of the methods conditions

#### 3.1.1. Influence of pH

**Fig. 5:**
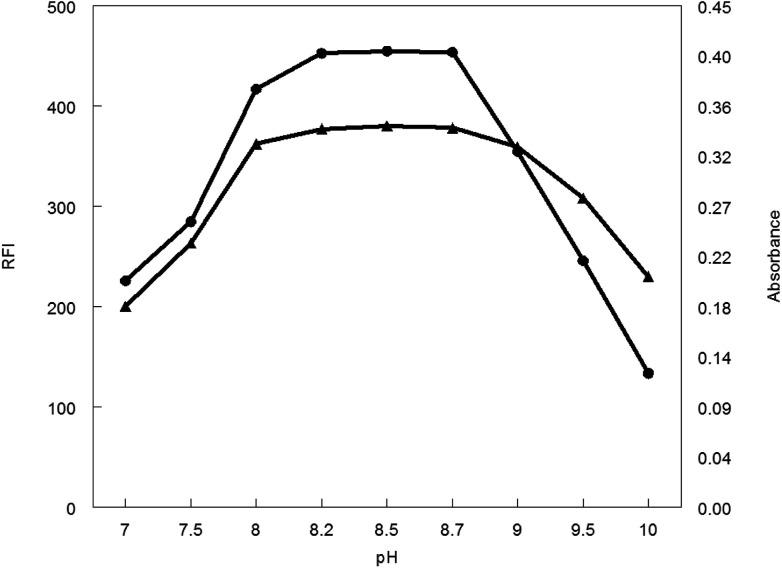
Effect of pH on RFI (▲) and absorbance (●) of the reaction product of MCH (0.2 and 9.0 μg mL^−1^, respectively) and NBD-Cl.

The influence of pH values of borate buffer (7–10) was examined to see its effect on the absorbance and fluorescence intensity of the reaction product. It was observed that, pH has a great effect on both absorbance and fluorescence intensity. The large value of the absorbance and fluorescence intensity was observed at pH range 8.2–8.7. Above or lower this pH range led to a decrease in the absorbance and fluorescence intensity (Fig. 5).

#### 3.1.2. Influence of buffer volume

**Fig. 6:**
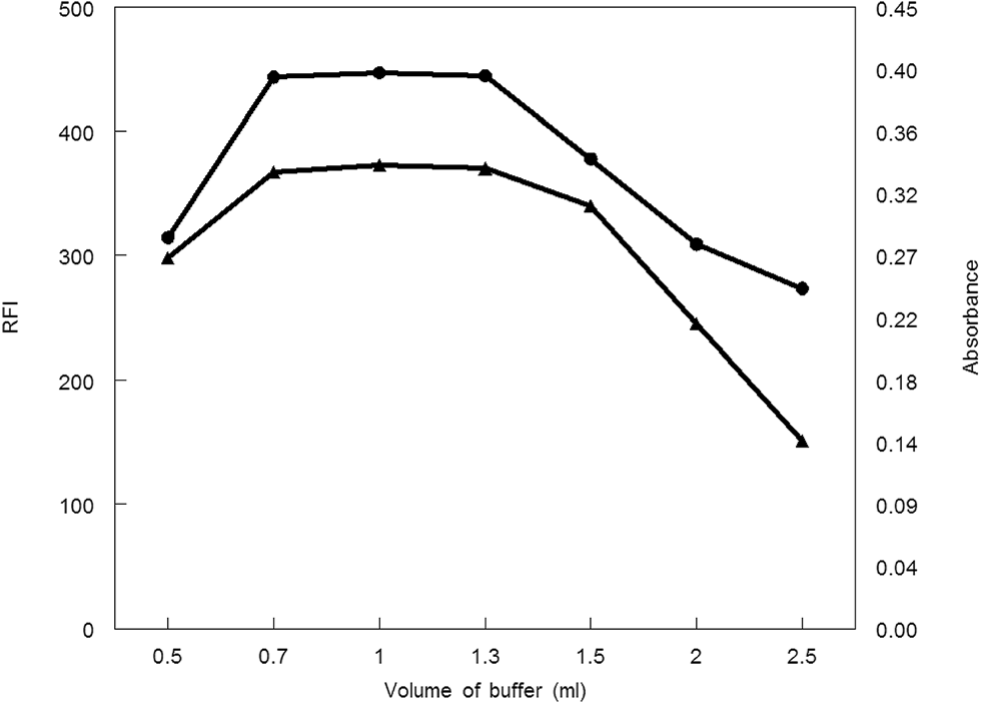
Effect of buffer volume on RFI (▲) and absorbance (●) of the reaction product of MCH (0.2 and 9.0 μg mL^−1^, respectively) and NBD-Cl.

Borate buffer (pH 8.5) with different volumes was applied to generate the analytical procedure for both methods. Maximum absorbance and fluorescence intensity was obtained in the volume range of 0.7–1.3 mL. Absorbance or fluorescence intensity was decreased above or below this volume range, thus 1 mL of borate buffer was preferable volume for other measurements (Fig. 6.).

#### 3.1.3. Influence of NBD-Cl volume

**Fig. 7:**
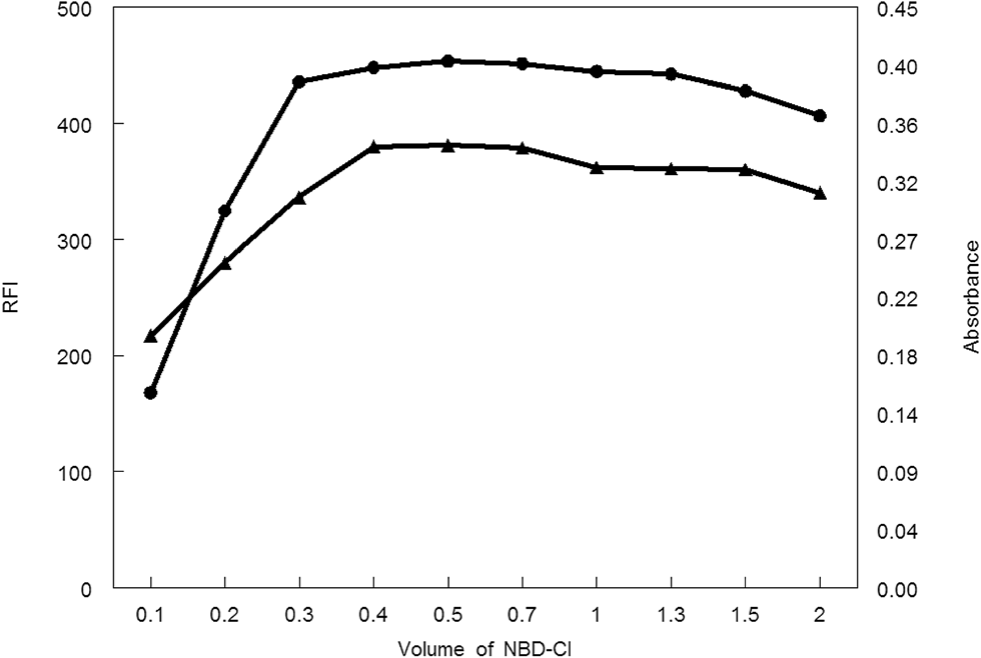
Effect of volume of NBD-Cl on RFI (▲) and absorbance (●) of the reaction product of MCH (0.2 and 9.0 μg mL^−1^, respectively) and NBD-Cl.

Various volumes of 0.1% w/v NBD-Cl were tried in carrying out the general analytical procedures. As shown in Fig. 7, the absorbance or fluorescence intensity was increased gradually upon increasing the reagent volume. A steady state was reached at 0.4–0.7 mL of NBD-Cl after which any additional raise of the NBD-Cl volume showed a decrease in the absorbance or fluorescence intensity. Consequently, 0.5 mL NBD-Cl was the recommended volume of the reagent for carrying out the general analytical procedure for both spectroscopic methods.

#### 3.1.4. Influence of warming period and temperature

One of the most important factors that can enhance or decrease the response of the reaction product (absorbance or fluorescence) are temperature at which reaction performed and heating time. In order to determine the optimum temperature and time, the derivatization reaction was carried out at room temperature (25 ± 5 °C) and the induced absorbance and fluorescence values were monitored at different time intervals. It was found that the reaction was very slow, and did not go to completion in reasonable time; it required more than 1 h. Therefore, investigations were carried out at varying elevated temperatures (40–100 °C), and the intensities of the induced absorbance and fluorescence were monitored at different time intervals. It was observed that, the absorbance or fluorescence intensity of the reaction product could be increased greatly either by elevating the reaction temperature or heating for a longer time. But, the main facing problem of these conditions is lacking of the reproducibility of the results, so we prefer heating the reaction content at a relatively low temperature (70 °C) for a longer heating time (20 min) than heating at a higher temperature for a shorter period of time to obtain reproducible results.

#### 3.1.5. Influence of diluting solvent

The last factor that can greatly affect the absorbance or fluorescence intensity of the reaction product was the type of the diluent. Distinctive diluents for instance (acetonitrile, distilled water, ethanol, acetone, and methanol) were tested to dilute the formed reaction product. From all of the investigated diluents, acetone was found to be the preferable diluent for dilution the reaction mixture as it accomplishes the greatest value of absorbance or fluorescence intensity.

### 3.2. Method validation

Validation of the presented spectroscopic methods was done according to ICH rules^20^ these parameters include.

#### 3.2.1. Linearity and range

Standard calibration plot was developed for the proposed spectrophotometric and spectrofluorimetric methods by plotting the relation between the absorbance and relative fluorescence intensity with the standard MCH concentrations. It was found that, absorbance at *λ*_max_ 465 nm and relative fluorescence intensity was linear with MCH concentration over the ranges of 1.5–12 and 0.03–0.5 μg mL^−1^ for the spectrophotometric and spectrofluorimetric methods, respectively. Quantification limits of the proposed methods were 1.09 and 0.02 μg mL^−1^ for the spectrophotometric and spectrofluorimetric methods, respectively. Other different statistical parameters of the proposed spectroscopic methods were listed in Table 1.

**Table 1:** Statistical parameters for the proposed spectroscopic methods for determination of MCH

Parameters	Spectrophotometric method	Spectrofluorimetric method
Wavelength	*λ*_max_ 465 nm	*λ*_ex_ 465 nm and *λ*_em_ 510 nm
Linearity range (μg mL^−1^)	1.5–12	0.03–0.5
Correlation coefficient (*r*)	0.9995	0.9998
Determination coefficient (*r*^2^)	0.9990	0.9996
Intercept ± SD	0.0771 ± 0.0039	89.109 ± 2.649
Slope ± SD	0.0355 ± 0.0005	1159.2 ± 10.036
LOD	0.36	0.007
LOQ	1.09	0.022

#### 3.2.2. Accuracy and precision

Accuracy of the proposed methods means the closeness of the measured values by the proposed spectroscopic methods to the true value and this was tested at five concentration levels within the recommended range. Each concentration level was measured three times and percent recovery ± standard deviation was calculated and listed in Table 2. Regarding the precisions (inter-day and intra-day),^20^ three different concentrations of the standard MCH found inside the linearity of each method were analyzed in different progressive days. The RSD values for intra-day precision was found to be less than 0.85 and 1.34 and inter-day precision was found to be less than 2.05 and 0.98, for the spectrophotometric and spectrofluorimetric methods, respectively, indicating the precision of the methods. The obtained results are tabulated in Table 3.

**Table 2:** Evaluation of accuracy of the proposed spectroscopic methods at five concentration levels within the linear range

Sample number	Spectrophotometric method		Spectrofluorimetric method	
	Conc. level (μg mL^−1^)	% Recovery^a^ ± SD	Conc. level (μg mL^−1^)	% Recovery^a^ ± SD
1	1.5	98.73 ± 1.28	0.1	98.47 ± 1.31
2	3	97.84 ± 1.13	0.2	100.96 ± 1.83
3	5	100.47 ± 0.92	0.3	98.37 ± 1.92
4	7	98.45 ± 0.94	0.4	99.23 ± 2.19
5	9	101.09 ± 1.57	0.5	99.26 ± 2.14

a The value is the average of three determinations.

**Table 3:** The intra- and inter-day precision for the determination of MCH by the proposed spectroscopic methods

Method	Amount^a^	% Recovery^b^ ± % RSD	
		Intra-day precision	Inter-day precision
Spectrophotometric method	3	100.81 ± 0.85	101.58 ± 2.05
	5	99.04 ± 0.61	98.56 ± 0.56
	7	98.20 ± 0.31	99.89 ± 1.19
Spectrofluorimetric method	0.1	99.80 ± 0.86	99.86 ± 0.62
	0.3	98.15 ± 1.34	99.97 ± 0.98
	0.5	100.85 ± 1.11	100.01 ± 0.82

a The amount is μg mL^−1^ for proposed methods.

b The value is the average of three determinations.

#### 3.2.3. Quantification and detection limits

LOD and LOQ are calculated adopting this equation “*x* = *n σ*/*S*”; where *x* is a symbol for LOD or LOQ, *n* is a numerical value equals 3.3 or 10 for LOD or LOQ, respectively, *σ* is the standard deviation of intercept and *S* is the slope.^20^ The estimated values for LOQ were 1.09 and 0.02 μg mL^−1^, for the spectrophotometric and spectrofluorimetric methods, respectively.

#### 3.2.4. Robustness

In our experimental procedures for the proposed methods, we made a small and deliberate change in the methods parameters to inspect the robustness of the spectroscopic methods and the relative standard deviations of the results were calculated. Variations in the proposed methods were carried out for the following three experimental parameters: NBD-Cl volume, solution pH, and volume of the buffer. The obtained data listed in Table 4 showed that the calculated RSD did not exceed 2.5%, which is a good sign for the acceptable robustness of the spectroscopic methods.

**Table 4:** Robustness of the proposed spectroscopic methods

Method parameters	Spectrophotometric method	Spectrofluorimetric method
	(%) Recovery^a^ ± % RSD	(%) Recovery^a^ ± % RSD
**Volume of NBD-Cl**		
0.4	98.95 ± 1.14	98.39 ± 0.63
0.7	98.84 ± 0.99	99.26 ± 1.22

**Buffer solution (pH)**		
8.2	100.71 ± 1.28	99.12 ± 0.72
8.7	98.81 ± 1.54	97.95 ± 1.18

**Volume of buffer**		
0.7	101.91 ± 0.96	100.14 ± 0.82
1.3	98.56 ± 1.51	99.69 ± 0.39

a Average of five determinations.

#### 3.2.5. Selectivity and specificity

The selectivity of the proposed methods was investigated by observing any interference encountered from the diverse sample matrix of the tablets, urine and plasma. The drug was analyzed in the presence of common tablet excipients such as starch, talc, mannitol, lactose and magnesium stearate. It was found that the presence of these excipients did not interfere with the proposed methods and this was proved by the excellent recoveries obtained (Table 5). Also, to ensure that the components of urine and plasma matrix did not interfere with the proposed spectrofluorimetric method, a blank experiment was carried out by applying the same procedures on urine and plasma sample free from the studied drug. It was found that urine and plasma matrix did not have any significant fluorescence intensity at the specified proposed method conditions (ESI Fig. 1†).

**Table 5:** Analysis of milnacipran (2 and 0.5 μg mL^−1^) in presence of some common excipients using the proposed spectrophotometric and spectrofluorimetric method, respectively

Excipients	Amount added (mg)	Spectrophotometric	Spectrofluorimetric
		(%) Recovery^a^ ± SD	(%) Recovery^a^± SD
Starch	100	97.18 ± 1.46	100.36 ± 0.65
Mg stearate	10	98.08 ± 0.46	99.53 ± 1.87
Talc	10	101.29 ± 0.63	10 020 ± 0.92
Lactose	10	99.80 ± 2.03	98.26 ± 1.83
Mannitol	10	99.27 ± 1.49	99.15 ± 1.89

a The value is the average of three determinations.

### 3.3. Application of the spectroscopic methods for assay of Avermilan® tablets

Assay of MCH in Avermilan® tablets was carried out by applying the proposed methods after their development and validation. The percentage mean recoveries value was 99.27 ± 1.18 and 99.44 ± 0.69 for the spectrophotometric and spectrofluorimetric methods, respectively as shown in Table 6. The results acquired from the two proposed methods and reference method^5^ were statistically compared regarding *t*- and *F*-tests at 95% confidence level. As the calculated *t*- and *F*-values are less than the tabulated one which proves the absence of any critical difference between the results of both methods regarding to the accuracy and precision.

**Table 6:** Results of the analysis of Averomilan® tablets containing milnacipran hydrochloride (50 mg for each tablet) using the proposed spectroscopic methods and reference method

Method	% Recovery^a^ ± SD		*t*-value^b^	*F*-value^b^
	Proposed method	Reference method^5^		
Spectrophotometric	99.27 ± 1.18	100.62 ± 1.26	1.736	1.132
Spectrofluorimetric	99.44 ± 0.69	100.62 ± 1.26	1.819	3.319

a Average of five determinations.

b Tabulated values for *t* and *F* are 2.306 and 6.338 respectively.

### 3.4. Application of the spectrofluorimetric method for assay of MCH in spiked human urine and plasma

It was reported about the pharmacokinetics of MCH that, oral administration of MCH tablet (50 mg) producing peak plasma concentration of was 0.135 mg L^−1^ after 2.0 hours and 50 to 60% of the drug is excreted unchanged in urine.^21^ The low value of LOQ (22 ng mL^−1^) of spectrofluorimetric method reflects its high sensitivity that enables us to assay of MCH in real and spiked human plasma. The high estimated mean recovery which listed in Table 7 indicates the appropriateness of the spectrofluorimetric method as preliminary *in vitro* study for the assay of MCH in human urine and plasma.

**Table 7:** Analysis of milnacipran hydrochloride in spiked human urine and plasma using the proposed spectrofluorimetric method

Concentration added (μg mL^−1^)	Urine	Plasma
	% Recovery^a^ ± SD	% Recovery^a^ ± SD
0.1	101.22 ± 2.04	101.97 ± 0.94
0.3	100.69 ± 1.54	98.83 ± 0.95
0.5	102.65 ± 1.03	100.34 ± 0.66
Mean ± SD	101.52 ± 1.01	100.38 ± 1.57
% RSD	0.99	1.56

a Average of three determinations.

## 4. Conclusion

In the present work, the chromogenic and fluorogenic properties of NBD-Cl were utilized for the development of two reproducible, simple, very sensitive and low cost spectroscopic methods for determination of the cited drug in pure form and in its tablets. Spectrofluorimetric method was considered to be the first spectrofluorimetric method for determination of MCH. The proposed spectroscopic methods were successfully applied for determination of MCH in its tablets with high percentage recoveries without excipients interference. Furthermore, spectrofluorimetric method was utilized as preliminary *in vitro* study for assay of MCH in human urine and plasma. The previously offered attractive merits by the proposed methods make our developed methods most recommended methods in quality control laboratories for determination of MCH.

## Conflicts of interest

There are no conflicts to declare.

## Supplementary Material

RA-008-C8RA03614D-s001
