# Supplementary material for: Utility of the chromogenic and fluorogenic properties of benzofurazan for the assay of milnacipran in human urine and plasma
Source: RSC Adv. 2018 Jun 15;8(39):22154–60. doi: 10.1039/c8ra03614d (PMC9081200; doi:10.1039/c8ra03614d)
Supplement: RA-008-C8RA03614D-s001 [file RA-008-C8RA03614D-s001.pdf]

Electronic Supplementary Material (ESI) for RSC Advances.
This journal is © The Royal Society of Chemistry 2018

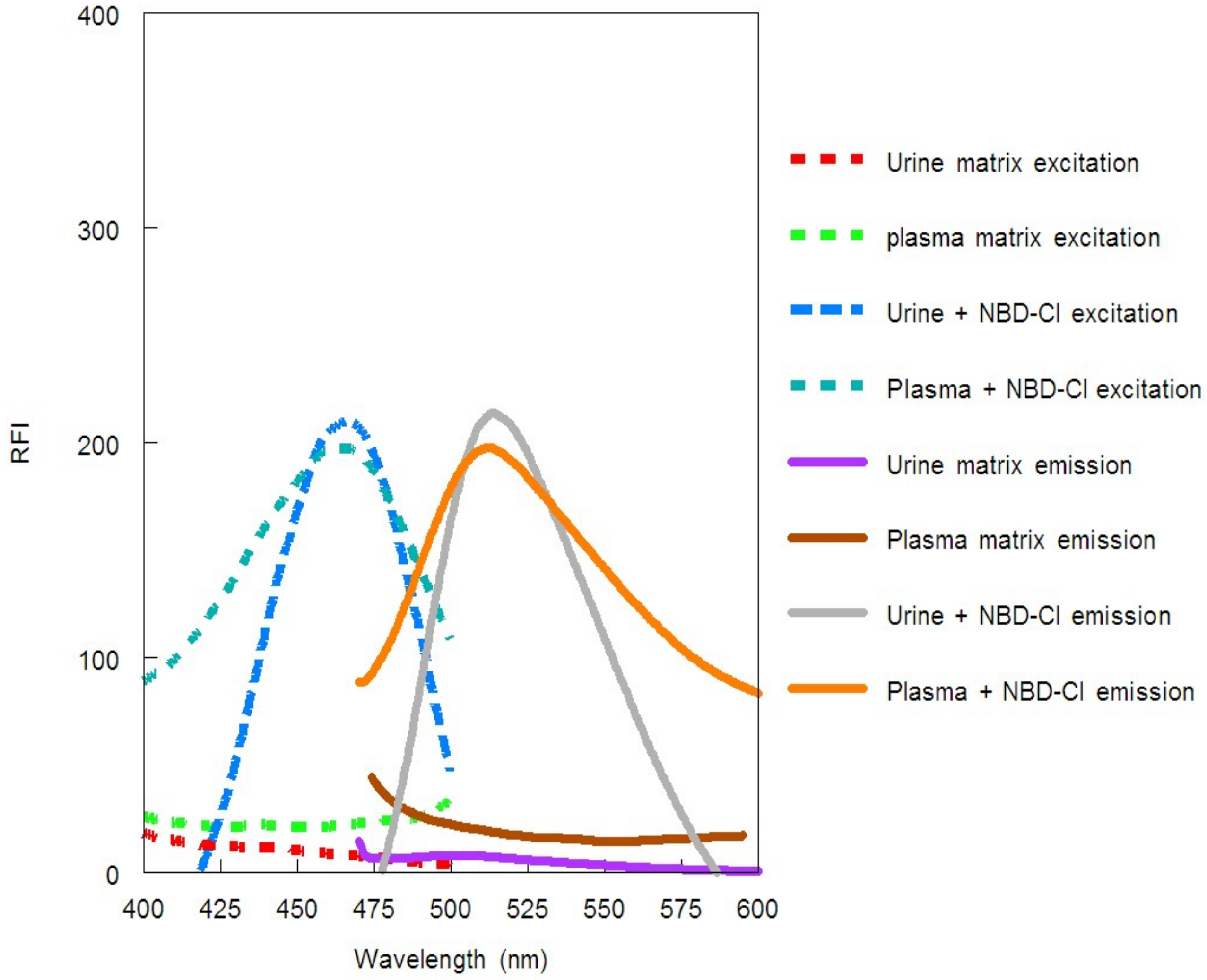

Fig 1 Fluorescence spectra of drug-free human urine and plasma using the general analytical procedure of the proposed spectrofluorimetric method

It should be noted that, the fluorescence intensity of the urine and plasma matrix with benzofurazan (NBD-Cl) is due to the NBD-Cl reagent background not the urine or plasma matrix components as NBD-Cl converted to NBD-OH in the alkaline medium of the borate buffer and we decreased this background in our methods by acidifying NBD-OH with hydrochloric acid.
